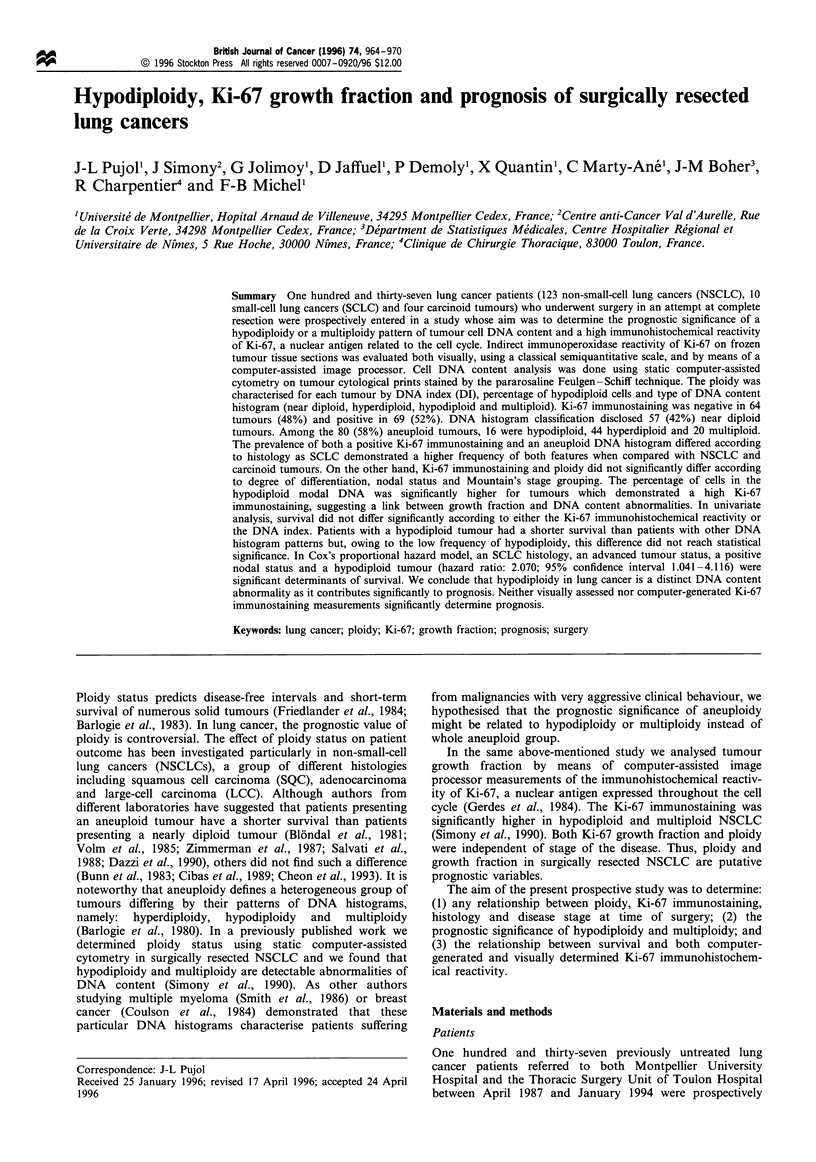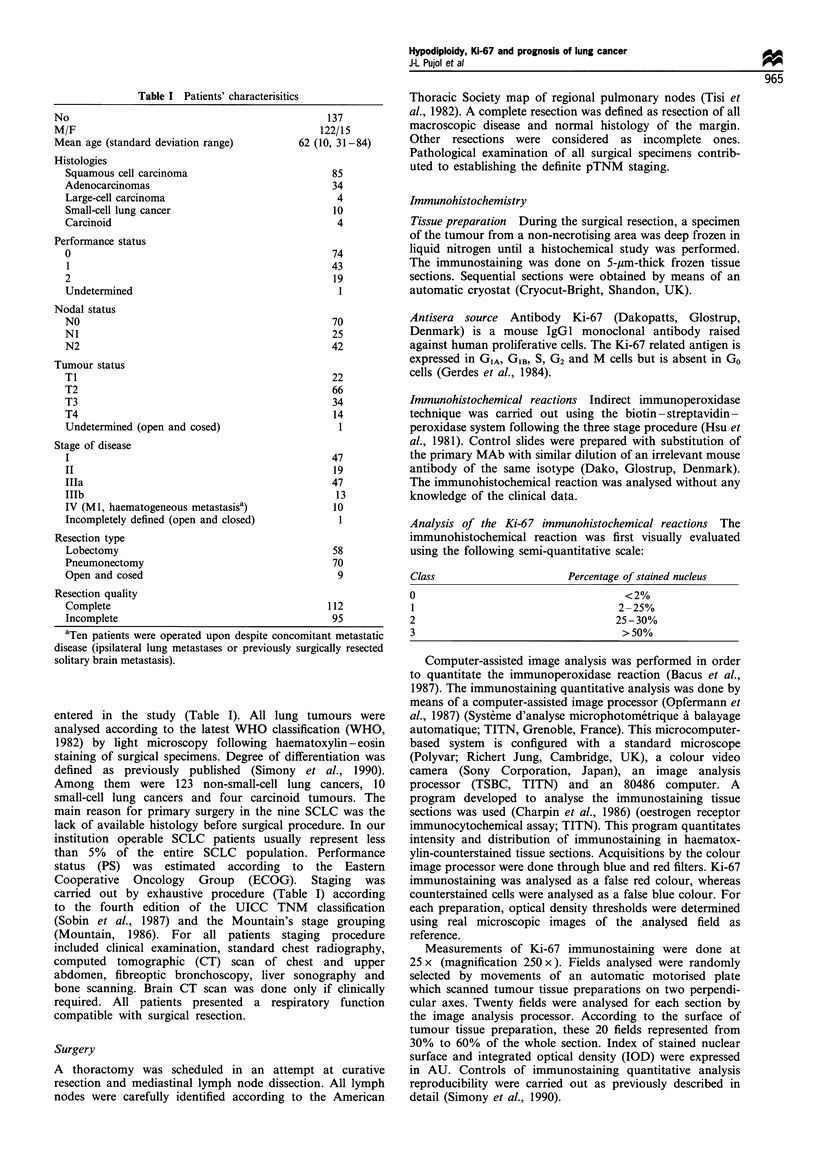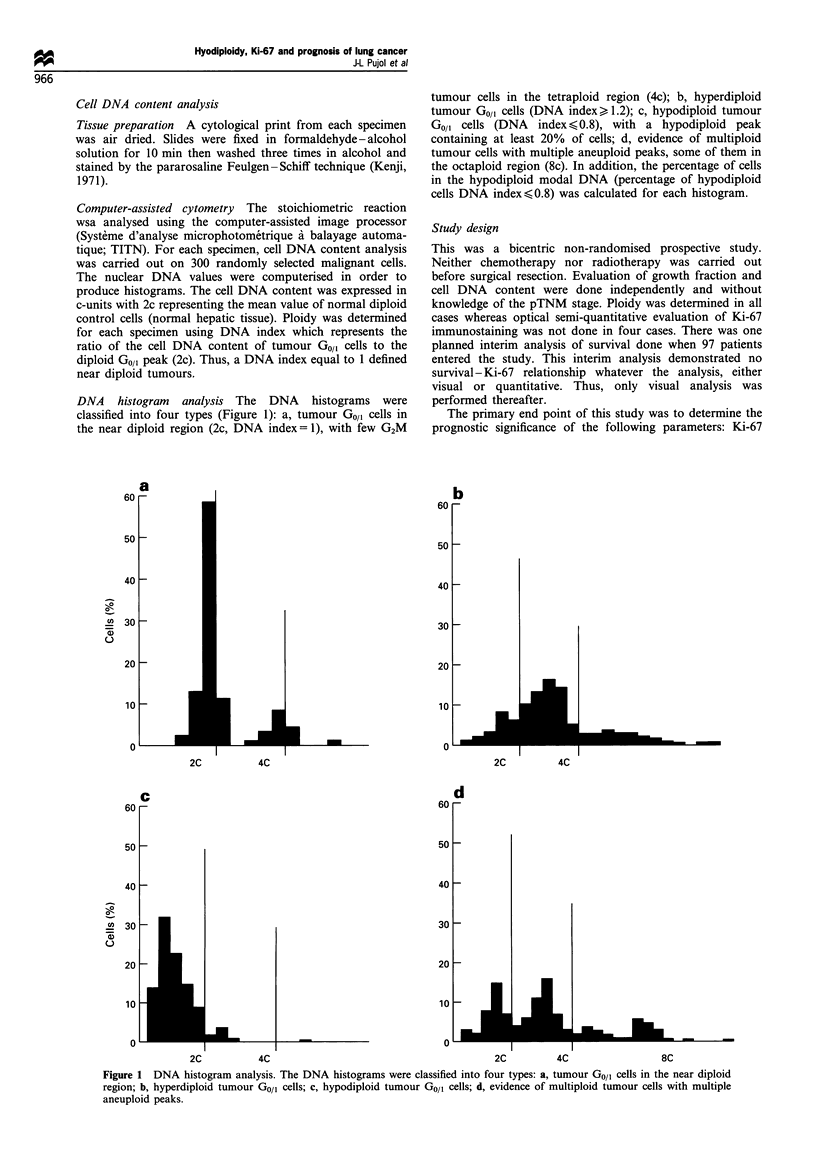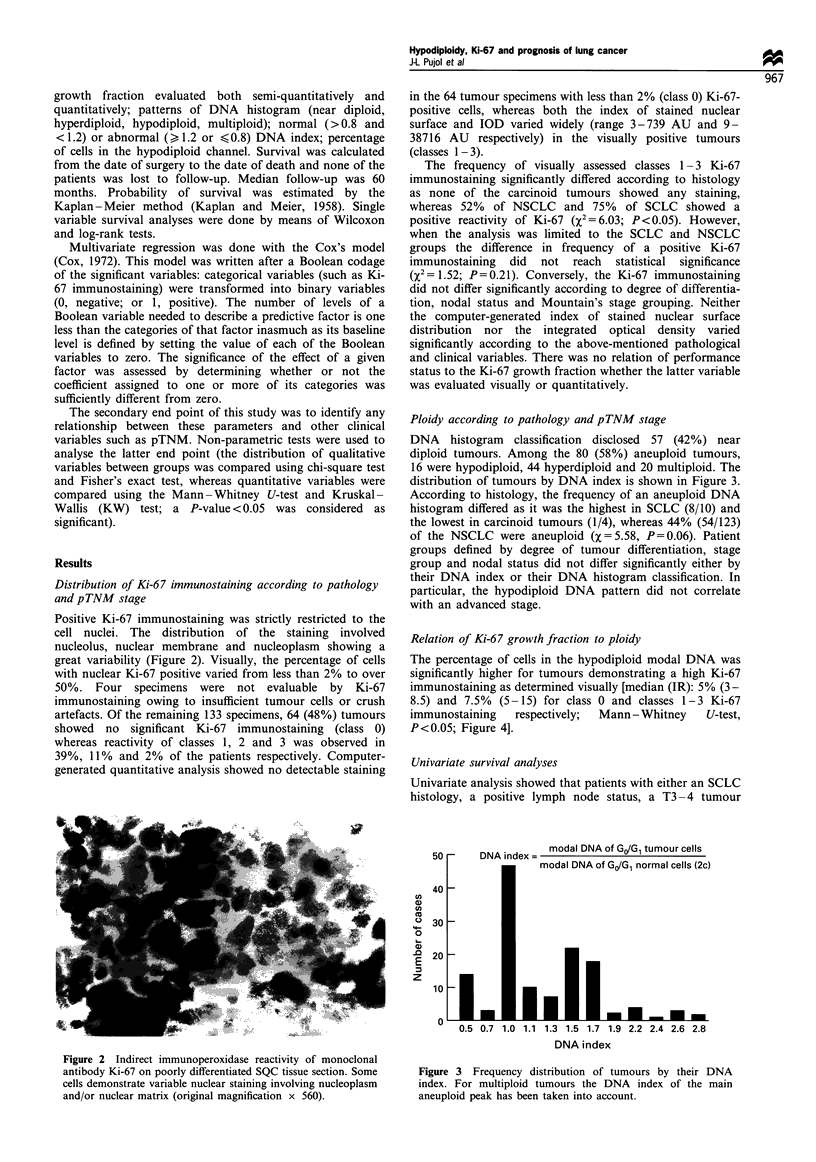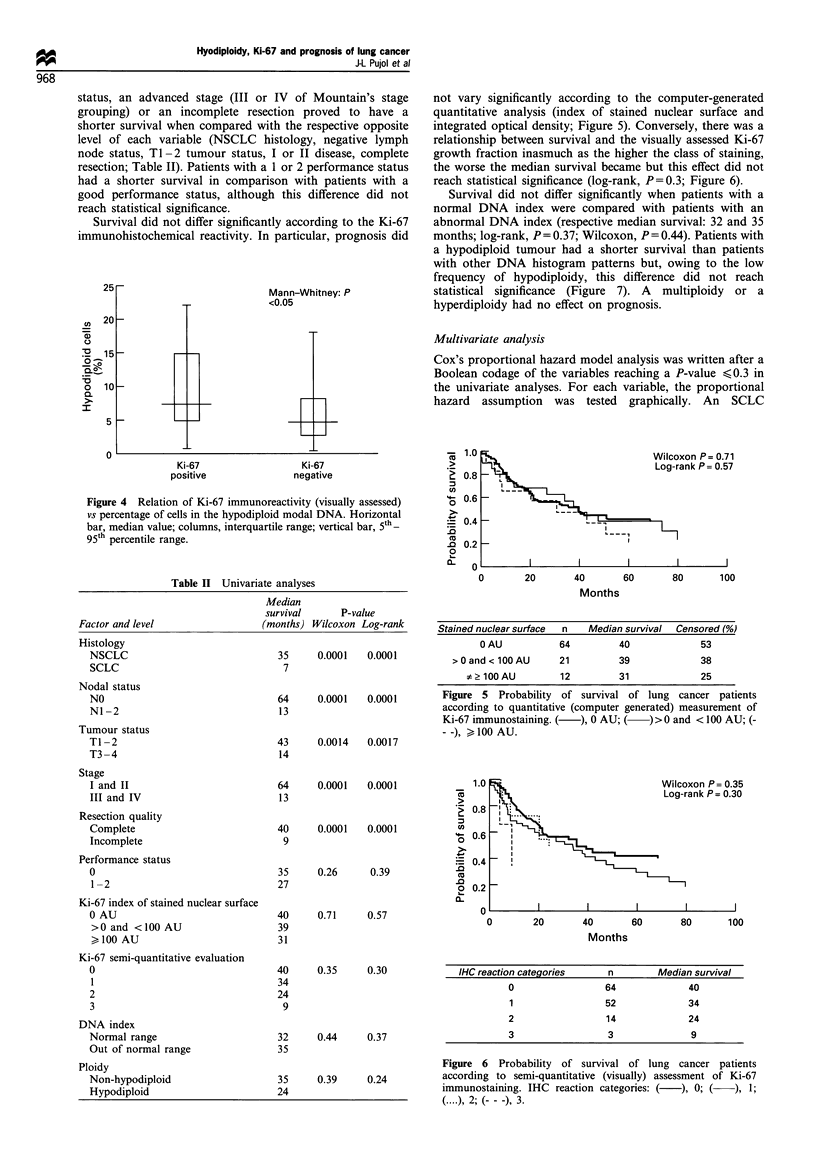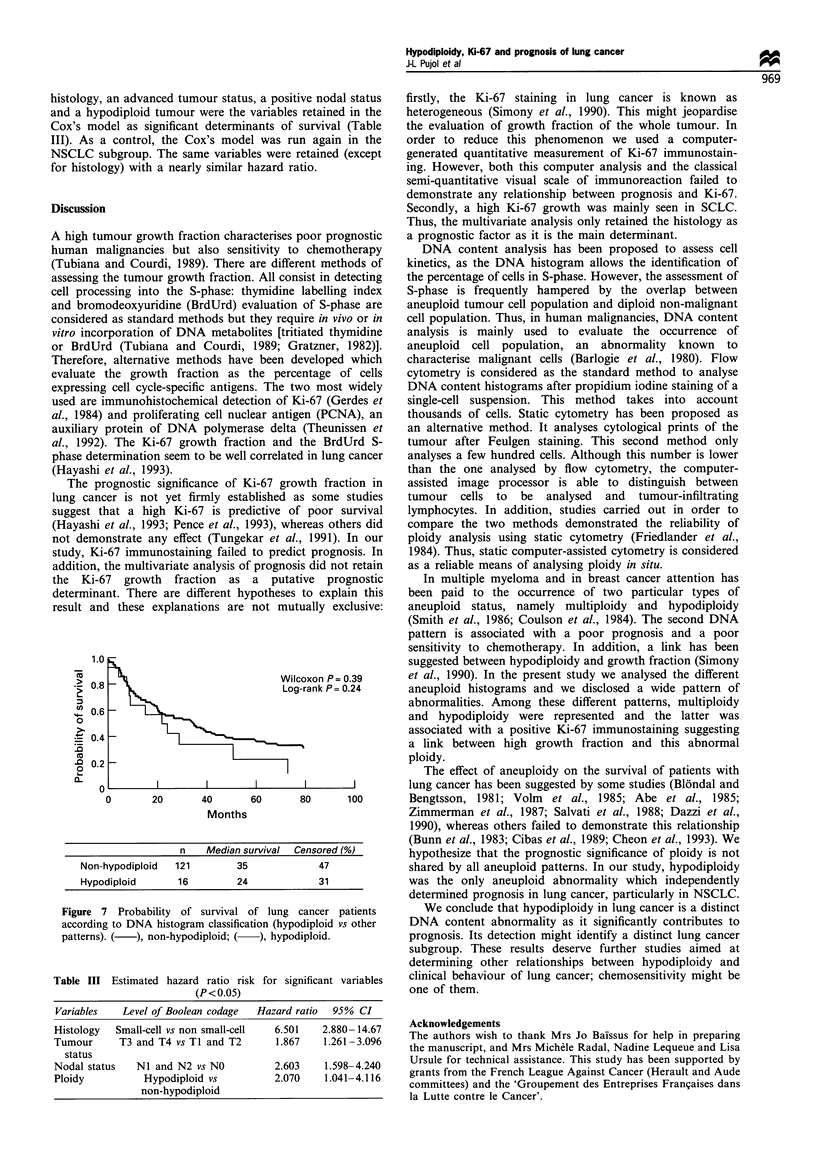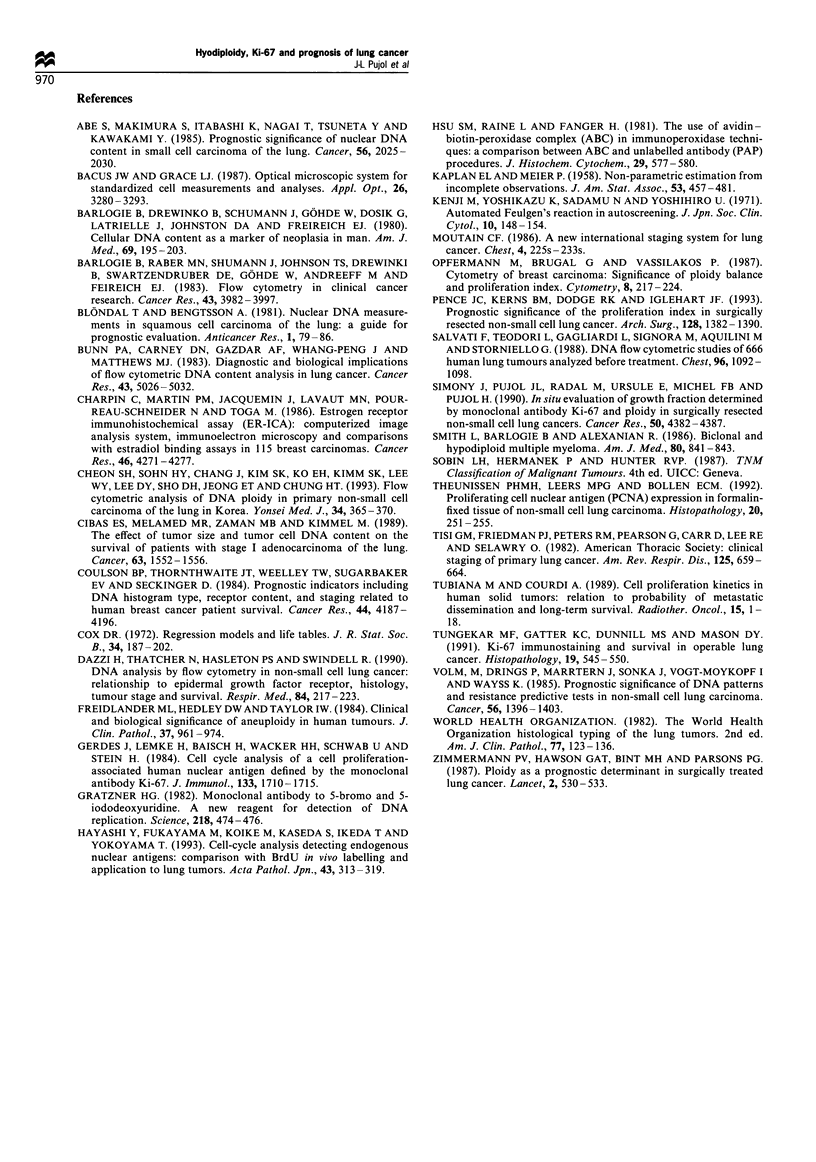# Hypodiploidy, Ki-67 growth fraction and prognosis of surgically resected lung cancers.

**DOI:** 10.1038/bjc.1996.466

**Published:** 1996-09

**Authors:** J. L. Pujol, J. Simony, G. Jolimoy, D. Jaffuel, P. Demoly, X. Quantin, C. Marty-Ané, J. M. Boher, R. Charpentier, F. B. Michel

**Affiliations:** Université de Montpellier, Hopital Arnaud de Villeneuve, France.

## Abstract

**Images:**


British Journal of Cancer (1996) 74, 964-970
© 1996 Stockton Press All rights reserved 0007-0920/96 $12.00

### Hypodiploidy, Ki-67 growth fraction and prognosis of surgically resected lung cancers

J-L Pujol[1], J Simony[2], G Jolimoy[1], D Jaffuel[1], P Demoly[1], X Quantin[1], C Marty-Ané[1], J-M Boher[3], R Charpentier[4] and F-B Michel[1]

*[1]Université de Montpellier, Hopital Arnaud de Villeneuve, 34295 Montpellier Cedex, France; [2]Centre anti-Cancer Val d'Aurelle, Rue de la Croix Verte, 34298 Montpellier Cedex, France; [3]Départment de Statistiques Médicales, Centre Hospitalier Régional et Universitaire de Nîmes, 5 Rue Hoche, 30000 Nîmes, France; [4]Clinique de Chirurgie Thoracique, 83000 Toulon, France.*

**Summary** One hundred and thirty-seven lung cancer patients (123 non-small-cell lung cancers (NSCLC), 10 small-cell lung cancers (SCLC) and four carcinoid tumours) who underwent surgery in an attempt at complete resection were prospectively entered in a study whose aim was to determine the prognostic significance of a hypodiploidy or a multiploidy pattern of tumour cell DNA content and a high immunohistochemical reactivity of Ki-67, a nuclear antigen related to the cell cycle. Indirect immunoperoxidase reactivity of Ki-67 on frozen tumour tissue sections was evaluated both visually, using a classical semiquantitative scale, and by means of a computer-assisted image processor. Cell DNA content analysis was done using static computer-assisted cytometry on tumour cytological prints stained by the pararosaline Feulgen-Schiff technique. The ploidy was characterised for each tumour by DNA index (DI), percentage of hypodiploid cells and type of DNA content histogram (near diploid, hyperdiploid, hypodiploid and multiploid). Ki-67 immunostaining was negative in 64 tumours (48%) and positive in 69 (52%). DNA histogram classification disclosed 57 (42%) near diploid tumours. Among the 80 (58%) aneuploid tumours, 16 were hypodiploid, 44 hyperdiploid and 20 multiploid. The prevalence of both a positive Ki-67 immunostaining and an aneuploid DNA histogram differed according to histology as SCLC demonstrated a higher frequency of both features when compared with NSCLC and carcinoid tumours. On the other hand, Ki-67 immunostaining and ploidy did not significantly differ according to degree of differentiation, nodal status and Mountain's stage grouping. The percentage of cells in the hypodiploid modal DNA was significantly higher for tumours which demonstrated a high Ki-67 immunostaining, suggesting a link between growth fraction and DNA content abnormalities. In univariate analysis, survival did not differ significantly according to either the Ki-67 immunohistochemical reactivity or the DNA index. Patients with a hypodiploid tumour had a shorter survival than patients with other DNA histogram patterns but, owing to the low frequency of hypodiploidy, this difference did not reach statistical significance. In Cox's proportional hazard model, an SCLC histology, an advanced tumour status, a positive nodal status and a hypodiploid tumour (hazard ratio: 2.070; 95% confidence interval 1.041-4.116) were significant determinants of survival. We conclude that hypodiploidy in lung cancer is a distinct DNA content abnormality as it contributes significantly to prognosis. Neither visually assessed nor computer-generated Ki-67 immunostaining measurements significantly determine prognosis.

**Keywords:** lung cancer; ploidy; Ki-67; growth fraction; prognosis; surgery

Ploidy status predicts disease-free intervals and short-term survival of numerous solid tumours (Friedlander *et al.*, 1984; Barlogie *et al.*, 1983). In lung cancer, the prognostic value of ploidy is controversial. The effect of ploidy status on patient outcome has been investigated particularly in non-small-cell lung cancers (NSCLCs), a group of different histologies including squamous cell carcinoma (SQC), adenocarcinoma and large-cell carcinoma (LCC). Although authors from different laboratories have suggested that patients presenting an aneuploid tumour have a shorter survival than patients presenting a nearly diploid tumour (Blöndal *et al.*, 1981; Volm *et al.*, 1985; Zimmerman *et al.*, 1987; Salvati *et al.*, 1988; Dazzi *et al.*, 1990), others did not find such a difference (Bunn *et al.*, 1983; Cibas *et al.*, 1989; Cheon *et al.*, 1993). It is noteworthy that aneuploidy defines a heterogeneous group of tumours differing by their patterns of DNA histograms, namely: hyperdiploidy, hypodiploidy and multiploidy (Barlogie *et al.*, 1980). In a previously published work we determined ploidy status using static computer-assisted cytometry in surgically resected NSCLC and we found that hypodiploidy and multiploidy are detectable abnormalities of DNA content (Simony *et al.*, 1990). As other authors studying multiple myeloma (Smith *et al.*, 1986) or breast cancer (Coulson *et al.*, 1984) demonstrated that these particular DNA histograms characterise patients suffering from malignancies with very aggressive clinical behaviour, we hypothesised that the prognostic significance of aneuploidy might be related to hypodiploidy or multiploidy instead of whole aneuploid group.

In the same above-mentioned study we analysed tumour growth fraction by means of computer-assisted image processor measurements of the immunohistochemical reactivity of Ki-67, a nuclear antigen expressed throughout the cell cycle (Gerdes *et al.*, 1984). The Ki-67 immunostaining was significantly higher in hypodiploid and multiploid NSCLC (Simony *et al.*, 1990). Both Ki-67 growth fraction and ploidy were independent of stage of the disease. Thus, ploidy and growth fraction in surgically resected NSCLC are putative prognostic variables.

The aim of the present prospective study was to determine: (1) any relationship between ploidy, Ki-67 immunostaining, histology and disease stage at time of surgery; (2) the prognostic significance of hypodiploidy and multiploidy; and (3) the relationship between survival and both computer-generated and visually determined Ki-67 immunohistochemical reactivity.

### Materials and methods

### *Patients*

One hundred and thirty-seven previously untreated lung cancer patients referred to both Montpellier University Hospital and the Thoracic Surgery Unit of Toulon Hospital between April 1987 and January 1994 were prospectively

Correspondence: J-L Pujol
Received 25 January 1996; revised 17 April 1996; accepted 24 April 1996

**Table I** Patients' characterisitics

| | |
|---|---|
| No | 137 |
| M/F | 122/15 |
| Mean age (standard deviation range) | 62 (10, 31–84) |
| Histologies | |
| Squamous cell carcinoma | 85 |
| Adenocarcinomas | 34 |
| Large-cell carcinoma | 4 |
| Small-cell lung cancer | 10 |
| Carcinoid | 4 |
| Performance status | |
| 0 | 74 |
| 1 | 43 |
| 2 | 19 |
| Undetermined | 1 |
| Nodal status | |
| N0 | 70 |
| N1 | 25 |
| N2 | 42 |
| Tumour status | |
| T1 | 22 |
| T2 | 66 |
| T3 | 34 |
| T4 | 14 |
| Undetermined (open and cosed) | 1 |
| Stage of disease | |
| I | 47 |
| II | 19 |
| IIIa | 47 |
| IIIb | 13 |
| IV (M1, haematogeneous metastasis[a]) | 10 |
| Incompletely defined (open and closed) | 1 |
| Resection type | |
| Lobectomy | 58 |
| Pneumonectomy | 70 |
| Open and cosed | 9 |
| Resection quality | |
| Complete | 112 |
| Incomplete | 95 |

[a]Ten patients were operated upon despite concomitant metastatic disease (ipsilateral lung metastases or previously surgically resected solitary brain metastasis).

entered in the study (Table I). All lung tumours were analysed according to the latest WHO classification (WHO, 1982) by light microscopy following haematoxylin–eosin staining of surgical specimens. Degree of differentiation was defined as previously published (Simony *et al.*, 1990). Among them were 123 non-small-cell lung cancers, 10 small-cell lung cancers and four carcinoid tumours. The main reason for primary surgery in the nine SCLC was the lack of available histology before surgical procedure. In our institution operable SCLC patients usually represent less than 5% of the entire SCLC population. Performance status (PS) was estimated according to the Eastern Cooperative Oncology Group (ECOG). Staging was carried out by exhaustive procedure (Table I) according to the fourth edition of the UICC TNM classification (Sobin *et al.*, 1987) and the Mountain's stage grouping (Mountain, 1986). For all patients staging procedure included clinical examination, standard chest radiography, computed tomographic (CT) scan of chest and upper abdomen, fibreoptic bronchoscopy, liver sonography and bone scanning. Brain CT scan was done only if clinically required. All patients presented a respiratory function compatible with surgical resection.

*Surgery*

A thoractomy was scheduled in an attempt at curative resection and mediastinal lymph node dissection. All lymph nodes were carefully identified according to the American Thoracic Society map of regional pulmonary nodes (Tisi *et al.*, 1982). A complete resection was defined as resection of all macroscopic disease and normal histology of the margin. Other resections were considered as incomplete ones. Pathological examination of all surgical specimens contributed to establishing the definite pTNM staging.

*Immunohistochemistry*

*Tissue preparation* During the surgical resection, a specimen of the tumour from a non-necrotising area was deep frozen in liquid nitrogen until a histochemical study was performed. The immunostaining was done on 5-$\mu$m-thick frozen tissue sections. Sequential sections were obtained by means of an automatic cryostat (Cryocut-Bright, Shandon, UK).

*Antisera source* Antibody Ki-67 (Dakopatts, Glostrup, Denmark) is a mouse IgG1 monoclonal antibody raised against human proliferative cells. The Ki-67 related antigen is expressed in $G_{1A}$, $G_{1B}$, S, $G_2$ and M cells but is absent in $G_0$ cells (Gerdes *et al.*, 1984).

*Immunohistochemical reactions* Indirect immunoperoxidase technique was carried out using the biotin–streptavidin–peroxidase system following the three stage procedure (Hsu *et al.*, 1981). Control slides were prepared with substitution of the primary MAb with similar dilution of an irrelevant mouse antibody of the same isotype (Dako, Glostrup, Denmark). The immunohistochemical reaction was analysed without any knowledge of the clinical data.

*Analysis of the Ki-67 immunohistochemical reactions* The immunohistochemical reaction was first visually evaluated using the following semi-quantitative scale:

| *Class* | *Percentage of stained nucleus* |
|---|---|
| 0 | <2% |
| 1 | 2–25% |
| 2 | 25–30% |
| 3 | >50% |

Computer-assisted image analysis was performed in order to quantitate the immunoperoxidase reaction (Bacus *et al.*, 1987). The immunostaining quantitative analysis was done by means of a computer-assisted image processor (Opfermann *et al.*, 1987) (Système d'analyse microphotométrique à balayage automatique; TITN, Grenoble, France). This microcomputer-based system is configured with a standard microscope (Polyvar; Richert Jung, Cambridge, UK), a colour video camera (Sony Corporation, Japan), an image analysis processor (TSBC, TITN) and an 80486 computer. A program developed to analyse the immunostaining tissue sections was used (Charpin *et al.*, 1986) (oestrogen receptor immunocytochemical assay; TITN). This program quantitates intensity and distribution of immunostaining in haematoxylin-counterstained tissue sections. Acquisitions by the colour image processor were done through blue and red filters. Ki-67 immunostaining was analysed as a false red colour, whereas counterstained cells were analysed as a false blue colour. For each preparation, optical density thresholds were determined using real microscopic images of the analysed field as reference.

Measurements of Ki-67 immunostaining were done at 25× (magnification 250×). Fields analysed were randomly selected by movements of an automatic motorised plate which scanned tumour tissue preparations on two perpendicular axes. Twenty fields were analysed for each section by the image analysis processor. According to the surface of tumour tissue preparation, these 20 fields represented from 30% to 60% of the whole section. Index of stained nuclear surface and integrated optical density (IOD) were expressed in AU. Controls of immunostaining quantitative analysis reproducibility were carried out as previously described in detail (Simony *et al.*, 1990).

*Cell DNA content analysis*

*Tissue preparation* A cytological print from each specimen was air dried. Slides were fixed in formaldehyde–alcohol solution for 10 min then washed three times in alcohol and stained by the pararosaline Feulgen–Schiff technique (Kenji, 1971).

*Computer-assisted cytometry* The stoichiometric reaction wsa analysed using the computer-assisted image processor (Système d'analyse microphotométrique à balayage automatique; TITN). For each specimen, cell DNA content analysis was carried out on 300 randomly selected malignant cells. The nuclear DNA values were computerised in order to produce histograms. The cell DNA content was expressed in c-units with 2c representing the mean value of normal diploid control cells (normal hepatic tissue). Ploidy was determined for each specimen using DNA index which represents the ratio of the cell DNA content of tumour $G_{0/1}$ cells to the diploid $G_{0/1}$ peak (2c). Thus, a DNA index equal to 1 defined near diploid tumours.

*DNA histogram analysis* The DNA histograms were classified into four types (Figure 1): a, tumour $G_{0/1}$ cells in the near diploid region (2c, DNA index = 1), with few $G_2M$ tumour cells in the tetraploid region (4c); b, hyperdiploid tumour $G_{0/1}$ cells (DNA index $\geqslant$ 1.2); c, hypodiploid tumour $G_{0/1}$ cells (DNA index $\leqslant$ 0.8), with a hypodiploid peak containing at least 20% of cells; d, evidence of multiploid tumour cells with multiple aneuploid peaks, some of them in the octaploid region (8c). In addition, the percentage of cells in the hypodiploid modal DNA (percentage of hypodiploid cells DNA index $\leqslant$ 0.8) was calculated for each histogram.

*Study design*

This was a bicentric non-randomised prospective study. Neither chemotherapy nor radiotherapy was carried out before surgical resection. Evaluation of growth fraction and cell DNA content were done independently and without knowledge of the pTNM stage. Ploidy was determined in all cases whereas optical semi-quantitative evaluation of Ki-67 immunostaining was not done in four cases. There was one planned interim analysis of survival done when 97 patients entered the study. This interim analysis demonstrated no survival–Ki-67 relationship whatever the analysis, either visual or quantitative. Thus, only visual analysis was performed thereafter.

The primary end point of this study was to determine the prognostic significance of the following parameters: Ki-67

**Figure 1** DNA histogram analysis. The DNA histograms were classified into four types: **a**, tumour $G_{0/1}$ cells in the near diploid region; **b**, hyperdiploid tumour $G_{0/1}$ cells; **c**, hypodiploid tumour $G_{0/1}$ cells; **d**, evidence of multiploid tumour cells with multiple aneuploid peaks.

growth fraction evaluated both semi-quantitatively and quantitatively; patterns of DNA histogram (near diploid, hyperdiploid, hypodiploid, multiploid); normal (>0.8 and <1.2) or abnormal (⩾1.2 or ⩽0.8) DNA index; percentage of cells in the hypodiploid channel. Survival was calculated from the date of surgery to the date of death and none of the patients was lost to follow-up. Median follow-up was 60 months. Probability of survival was estimated by the Kaplan–Meier method (Kaplan and Meier, 1958). Single variable survival analyses were done by means of Wilcoxon and log-rank tests.

Multivariate regression was done with the Cox's model (Cox, 1972). This model was written after a Boolean codage of the significant variables: categorical variables (such as Ki-67 immunostaining) were transformed into binary variables (0, negative; or 1, positive). The number of levels of a Boolean variable needed to describe a predictive factor is one less than the categories of that factor inasmuch as its baseline level is defined by setting the value of each of the Boolean variables to zero. The significance of the effect of a given factor was assessed by determining whether or not the coefficient assigned to one or more of its categories was sufficiently different from zero.

The secondary end point of this study was to identify any relationship between these parameters and other clinical variables such as pTNM. Non-parametric tests were used to analyse the latter end point (the distribution of qualitative variables between groups was compared using chi-square test and Fisher's exact test, whereas quantitative variables were compared using the Mann–Whitney *U*-test and Kruskal–Wallis (KW) test; a *P*-value<0.05 was considered as significant).

### Results

### *Distribution of Ki-67 immunostaining according to pathology and pTNM stage*

Positive Ki-67 immunostaining was strictly restricted to the cell nuclei. The distribution of the staining involved nucleolus, nuclear membrane and nucleoplasm showing a great variability (Figure 2). Visually, the percentage of cells with nuclear Ki-67 positive varied from less than 2% to over 50%. Four specimens were not evaluable by Ki-67 immunostaining owing to insufficient tumour cells or crush artefacts. Of the remaining 133 specimens, 64 (48%) tumours showed no significant Ki-67 immunostaining (class 0) whereas reactivity of classes 1, 2 and 3 was observed in 39%, 11% and 2% of the patients respectively. Computer-generated quantitative analysis showed no detectable staining in the 64 tumour specimens with less than 2% (class 0) Ki-67-positive cells, whereas both the index of stained nuclear surface and IOD varied widely (range 3–739 AU and 9–38716 AU respectively) in the visually positive tumours (classes 1–3).

The frequency of visually assessed classes 1–3 Ki-67 immunostaining significantly differed according to histology as none of the carcinoid tumours showed any staining, whereas 52% of NSCLC and 75% of SCLC showed a positive reactivity of Ki-67 ($\chi^2=6.03$; $P<0.05$). However, when the analysis was limited to the SCLC and NSCLC groups the difference in frequency of a positive Ki-67 immunostaining did not reach statistical significance ($\chi^2=1.52$; $P=0.21$). Conversely, the Ki-67 immunostaining did not differ significantly according to degree of differentiation, nodal status and Mountain's stage grouping. Neither the computer-generated index of stained nuclear surface distribution nor the integrated optical density varied significantly according to the above-mentioned pathological and clinical variables. There was no relation of performance status to the Ki-67 growth fraction whether the latter variable was evaluated visually or quantitatively.

### *Ploidy according to pathology and pTNM stage*

DNA histogram classification disclosed 57 (42%) near diploid tumours. Among the 80 (58%) aneuploid tumours, 16 were hypodiploid, 44 hyperdiploid and 20 multiploid. The distribution of tumours by DNA index is shown in Figure 3. According to histology, the frequency of an aneuploid DNA histogram differed as it was the highest in SCLC (8/10) and the lowest in carcinoid tumours (1/4), whereas 44% (54/123) of the NSCLC were aneuploid ($\chi=5.58$, $P=0.06$). Patient groups defined by degree of tumour differentiation, stage group and nodal status did not differ significantly either by their DNA index or their DNA histogram classification. In particular, the hypodiploid DNA pattern did not correlate with an advanced stage.

### *Relation of Ki-67 growth fraction to ploidy*

The percentage of cells in the hypodiploid modal DNA was significantly higher for tumours demonstrating a high Ki-67 immunostaining as determined visually [median (IR): 5% (3–8.5) and 7.5% (5–15) for class 0 and classes 1–3 Ki-67 immunostaining respectively; Mann–Whitney *U*-test, $P<0.05$; Figure 4].

### *Univariate survival analyses*

Univariate analysis showed that patients with either an SCLC histology, a positive lymph node status, a T3–4 tumour

**Figure 2** Indirect immunoperoxidase reactivity of monoclonal antibody Ki-67 on poorly differentiated SQC tissue section. Some cells demonstrate variable nuclear staining involving nucleoplasm and/or nuclear matrix (original magnification × 560).

**Figure 3** Frequency distribution of tumours by their DNA index. For multiploid tumours the DNA index of the main aneuploid peak has been taken into account.

status, an advanced stage (III or IV of Mountain's stage grouping) or an incomplete resection proved to have a shorter survival when compared with the respective opposite level of each variable (NSCLC histology, negative lymph node status, T1-2 tumour status, I or II disease, complete resection; Table II). Patients with a 1 or 2 performance status had a shorter survival in comparison with patients with a good performance status, although this difference did not reach statistical significance.

Survival did not differ significantly according to the Ki-67 immunohistochemical reactivity. In particular, prognosis did

**Figure 4** Relation of Ki-67 immunoreactivity (visually assessed) *vs* percentage of cells in the hypodiploid modal DNA. Horizontal bar, median value; columns, interquartile range; vertical bar, $5^{th}-95^{th}$ percentile range.

**Table II** Univariate analyses

| *Factor and level* | *Median survival (months)* | P-*value* *Wilcoxon* | *Log-rank* |
|---|---|---|---|
| Histology | | | |
| NSCLC | 35 | 0.0001 | 0.0001 |
| SCLC | 7 | | |
| Nodal status | | | |
| N0 | 64 | 0.0001 | 0.0001 |
| N1-2 | 13 | | |
| Tumour status | | | |
| T1-2 | 43 | 0.0014 | 0.0017 |
| T3-4 | 14 | | |
| Stage | | | |
| I and II | 64 | 0.0001 | 0.0001 |
| III and IV | 13 | | |
| Resection quality | | | |
| Complete | 40 | 0.0001 | 0.0001 |
| Incomplete | 9 | | |
| Performance status | | | |
| 0 | 35 | 0.26 | 0.39 |
| 1-2 | 27 | | |
| Ki-67 index of stained nuclear surface | | | |
| 0 AU | 40 | 0.71 | 0.57 |
| >0 and <100 AU | 39 | | |
| ⩾100 AU | 31 | | |
| Ki-67 semi-quantitative evaluation | | | |
| 0 | 40 | 0.35 | 0.30 |
| 1 | 34 | | |
| 2 | 24 | | |
| 3 | 9 | | |
| DNA index | | | |
| Normal range | 32 | 0.44 | 0.37 |
| Out of normal range | 35 | | |
| Ploidy | | | |
| Non-hypodiploid | 35 | 0.39 | 0.24 |
| Hypodiploid | 24 | | |

not vary significantly according to the computer-generated quantitative analysis (index of stained nuclear surface and integrated optical density; Figure 5). Conversely, there was a relationship between survival and the visually assessed Ki-67 growth fraction inasmuch as the higher the class of staining, the worse the median survival became but this effect did not reach statistical significance (log-rank, $P=0.3$; Figure 6).

Survival did not differ significantly when patients with a normal DNA index were compared with patients with an abnormal DNA index (respective median survival: 32 and 35 months; log-rank, $P=0.37$; Wilcoxon, $P=0.44$). Patients with a hypodiploid tumour had a shorter survival than patients with other DNA histogram patterns but, owing to the low frequency of hypodiploidy, this difference did not reach statistical significance (Figure 7). A multiploidy or a hyperdiploidy had no effect on prognosis.

### *Multivariate analysis*

Cox's proportional hazard model analysis was written after a Boolean codage of the variables reaching a *P*-value ⩽0.3 in the univariate analyses. For each variable, the proportional hazard assumption was tested graphically. An SCLC

| *Stained nuclear surface* | n | *Median survival* | *Censored (%)* |
|---|---|---|---|
| 0 AU | 64 | 40 | 53 |
| > 0 and < 100 AU | 21 | 39 | 38 |
| ≠ ≥ 100 AU | 12 | 31 | 25 |

**Figure 5** Probability of survival of lung cancer patients according to quantitative (computer generated) measurement of Ki-67 immunostaining. (——), 0 AU; (——)>0 and <100 AU; (- - -), ⩾100 AU.

| *IHC reaction categories* | n | *Median survival* |
|---|---|---|
| 0 | 64 | 40 |
| 1 | 52 | 34 |
| 2 | 14 | 24 |
| 3 | 3 | 9 |

**Figure 6** Probability of survival of lung cancer patients according to semi-quantitative (visually) assessment of Ki-67 immunostaining. IHC reaction categories: (——), 0; (——), 1; (....), 2; (- - -), 3.

histology, an advanced tumour status, a positive nodal status and a hypodiploid tumour were the variables retained in the Cox's model as significant determinants of survival (Table III). As a control, the Cox's model was run again in the NSCLC subgroup. The same variables were retained (except for histology) with a nearly similar hazard ratio.

### Discussion

A high tumour growth fraction characterises poor prognostic human malignancies but also sensitivity to chemotherapy (Tubiana and Courdi, 1989). There are different methods of assessing the tumour growth fraction. All consist in detecting cell processing into the S-phase: thymidine labelling index and bromodeoxyuridine (BrdUrd) evaluation of S-phase are considered as standard methods but they require *in vivo* or *in vitro* incorporation of DNA metabolites [tritiated thymidine or BrdUrd (Tubiana and Courdi, 1989; Gratzner, 1982)]. Therefore, alternative methods have been developed which evaluate the growth fraction as the percentage of cells expressing cell cycle-specific antigens. The two most widely used are immunohistochemical detection of Ki-67 (Gerdes *et al.*, 1984) and proliferating cell nuclear antigen (PCNA), an auxiliary protein of DNA polymerase delta (Theunissen *et al.*, 1992). The Ki-67 growth fraction and the BrdUrd S-phase determination seem to be well correlated in lung cancer (Hayashi *et al.*, 1993).

The prognostic significance of Ki-67 growth fraction in lung cancer is not yet firmly established as some studies suggest that a high Ki-67 is predictive of poor survival (Hayashi *et al.*, 1993; Pence *et al.*, 1993), whereas others did not demonstrate any effect (Tungekar *et al.*, 1991). In our study, Ki-67 immunostaining failed to predict prognosis. In addition, the multivariate analysis of prognosis did not retain the Ki-67 growth fraction as a putative prognostic determinant. There are different hypotheses to explain this result and these explanations are not mutually exclusive:

| | n | *Median survival* | *Censored (%)* |
|---|---|---|---|
| Non-hypodiploid | 121 | 35 | 47 |
| Hypodiploid | 16 | 24 | 31 |

**Figure 7** Probability of survival of lung cancer patients according to DNA histogram classification (hypodiploid *vs* other patterns). (——), non-hypodiploid; (——), hypodiploid.

**Table III** Estimated hazard ratio risk for significant variables ($P<0.05$)

| *Variables* | *Level of Boolean codage* | *Hazard ratio* | *95% CI* |
|---|---|---|---|
| Histology | Small-cell *vs* non small-cell | 6.501 | 2.880–14.67 |
| Tumour status | T3 and T4 *vs* T1 and T2 | 1.867 | 1.261–3.096 |
| Nodal status | N1 and N2 *vs* N0 | 2.603 | 1.598–4.240 |
| Ploidy | Hypodiploid *vs* non-hypodiploid | 2.070 | 1.041–4.116 |

firstly, the Ki-67 staining in lung cancer is known as heterogeneous (Simony *et al.*, 1990). This might jeopardise the evaluation of growth fraction of the whole tumour. In order to reduce this phenomenon we used a computer-generated quantitative measurement of Ki-67 immunostaining. However, both this computer analysis and the classical semi-quantitative visual scale of immunoreaction failed to demonstrate any relationship between prognosis and Ki-67. Secondly, a high Ki-67 growth was mainly seen in SCLC. Thus, the multivariate analysis only retained the histology as a prognostic factor as it is the main determinant.

DNA content analysis has been proposed to assess cell kinetics, as the DNA histogram allows the identification of the percentage of cells in S-phase. However, the assessment of S-phase is frequently hampered by the overlap between aneuploid tumour cell population and diploid non-malignant cell population. Thus, in human malignancies, DNA content analysis is mainly used to evaluate the occurrence of aneuploid cell population, an abnormality known to characterise malignant cells (Barlogie *et al.*, 1980). Flow cytometry is considered as the standard method to analyse DNA content histograms after propidium iodine staining of a single-cell suspension. This method takes into account thousands of cells. Static cytometry has been proposed as an alternative method. It analyses cytological prints of the tumour after Feulgen staining. This second method only analyses a few hundred cells. Although this number is lower than the one analysed by flow cytometry, the computer-assisted image processor is able to distinguish between tumour cells to be analysed and tumour-infiltrating lymphocytes. In addition, studies carried out in order to compare the two methods demonstrated the reliability of ploidy analysis using static cytometry (Friedlander *et al.*, 1984). Thus, static computer-assisted cytometry is considered as a reliable means of analysing ploidy *in situ*.

In multiple myeloma and in breast cancer attention has been paid to the occurrence of two particular types of aneuploid status, namely multiploidy and hypodiploidy (Smith *et al.*, 1986; Coulson *et al.*, 1984). The second DNA pattern is associated with a poor prognosis and a poor sensitivity to chemotherapy. In addition, a link has been suggested between hypodiploidy and growth fraction (Simony *et al.*, 1990). In the present study we analysed the different aneuploid histograms and we disclosed a wide pattern of abnormalities. Among these different patterns, multiploidy and hypodiploidy were represented and the latter was associated with a positive Ki-67 immunostaining suggesting a link between high growth fraction and this abnormal ploidy.

The effect of aneuploidy on the survival of patients with lung cancer has been suggested by some studies (Blöndal and Bengtsson, 1981; Volm *et al.*, 1985; Abe *et al.*, 1985; Zimmerman *et al.*, 1987; Salvati *et al.*, 1988; Dazzi *et al.*, 1990), whereas others failed to demonstrate this relationship (Bunn *et al.*, 1983; Cibas *et al.*, 1989; Cheon *et al.*, 1993). We hypothesize that the prognostic significance of ploidy is not shared by all aneuploid patterns. In our study, hypodiploidy was the only aneuploid abnormality which independently determined prognosis in lung cancer, particularly in NSCLC.

We conclude that hypodiploidy in lung cancer is a distinct DNA content abnormality as it significantly contributes to prognosis. Its detection might identify a distinct lung cancer subgroup. These results deserve further studies aimed at determining other relationships between hypodiploidy and clinical behaviour of lung cancer; chemosensitivity might be one of them.

**Acknowledgements**
The authors wish to thank Mrs Jo Baïssus for help in preparing the manuscript, and Mrs Michèle Radal, Nadine Lequeue and Lisa Ursule for technical assistance. This study has been supported by grants from the French League Against Cancer (Herault and Aude committees) and the 'Groupement des Entreprises Françaises dans la Lutte contre le Cancer'.

### References

ABE S, MAKIMURA S, ITABASHI K, NAGAI T, TSUNETA Y AND KAWAKAMI Y. (1985). Prognostic significance of nuclear DNA content in small cell carcinoma of the lung. *Cancer*, **56**, 2025–2030.

BACUS JW AND GRACE LJ. (1987). Optical microscopic system for standardized cell measurements and analyses. *Appl. Opt.*, **26**, 3280–3293.

BARLOGIE B, DREWINKO B, SCHUMANN J, GÖHDE W, DOSIK G, LATRIELLE J, JOHNSTON DA AND FREIREICH EJ. (1980). Cellular DNA content as a marker of neoplasia in man. *Am. J. Med.*, **69**, 195–203.

BARLOGIE B, RABER MN, SHUMANN J, JOHNSON TS, DREWINKI B, SWARTZENDRUBER DE, GÖHDE W, ANDREEFF M AND FEIREICH EJ. (1983). Flow cytometry in clinical cancer research. *Cancer Res.*, **43**, 3982–3997.

BLÖNDAL T AND BENGTSSON A. (1981). Nuclear DNA measurements in squamous cell carcinoma of the lung: a guide for prognostic evaluation. *Anticancer Res.*, **1**, 79–86.

BUNN PA, CARNEY DN, GAZDAR AF, WHANG-PENG J AND MATTHEWS MJ. (1983). Diagnostic and biological implications of flow cytometric DNA content analysis in lung cancer. *Cancer Res.*, **43**, 5026–5032.

CHARPIN C, MARTIN PM, JACQUEMIN J, LAVAUT MN, POURREAU-SCHNEIDER N AND TOGA M. (1986). Estrogen receptor immunohistochemical assay (ER-ICA): computerized image analysis system, immunoelectron microscopy and comparisons with estradiol binding assays in 115 breast carcinomas. *Cancer Res.*, **46**, 4271–4277.

CHEON SH, SOHN HY, CHANG J, KIM SK, KO EH, KIMM SK, LEE WY, LEE DY, SHO DH, JEONG ET AND CHUNG HT. (1993). Flow cytometric analysis of DNA ploidy in primary non-small cell carcinoma of the lung in Korea. *Yonsei Med. J.*, **34**, 365–370.

CIBAS ES, MELAMED MR, ZAMAN MB AND KIMMEL M. (1989). The effect of tumor size and tumor cell DNA content on the survival of patients with stage I adenocarcinoma of the lung. *Cancer*, **63**, 1552–1556.

COULSON BP, THORNTHWAITE JT, WEELLEY TW, SUGARBAKER EV AND SECKINGER D. (1984). Prognostic indicators including DNA histogram type, receptor content, and staging related to human breast cancer patient survival. *Cancer Res.*, **44**, 4187–4196.

COX DR. (1972). Regression models and life tables. *J. R. Stat. Soc. B.*, **34**, 187–202.

DAZZI H, THATCHER N, HASLETON PS AND SWINDELL R. (1990). DNA analysis by flow cytometry in non-small cell lung cancer: relationship to epidermal growth factor receptor, histology, tumour stage and survival. *Respir. Med.*, **84**, 217–223.

FREIDLANDER ML, HEDLEY DW AND TAYLOR IW. (1984). Clinical and biological significance of aneuploidy in human tumours. *J. Clin. Pathol.*, **37**, 961–974.

GERDES J, LEMKE H, BAISCH H, WACKER HH, SCHWAB U AND STEIN H. (1984). Cell cycle analysis of a cell proliferation-associated human nuclear antigen defined by the monoclonal antibody Ki-67. *J. Immunol.*, **133**, 1710–1715.

GRATZNER HG. (1982). Monoclonal antibody to 5-bromo and 5-iododeoxyuridine. A new reagent for detection of DNA replication. *Science*, **218**, 474–476.

HAYASHI Y, FUKAYAMA M, KOIKE M, KASEDA S, IKEDA T AND YOKOYAMA T. (1993). Cell-cycle analysis detecting endogenous nuclear antigens: comparison with BrdU *in vivo* labelling and application to lung tumors. *Acta Pathol. Jpn.*, **43**, 313–319.

HSU SM, RAINE L AND FANGER H. (1981). The use of avidin–biotin-peroxidase complex (ABC) in immunoperoxidase techniques: a comparison between ABC and unlabelled antibody (PAP) procedures. *J. Histochem. Cytochem.*, **29**, 577–580.

KAPLAN EL AND MEIER P. (1958). Non-parametric estimation from incomplete observations. *J. Am. Stat. Assoc.*, **53**, 457–481.

KENJI M, YOSHIKAZU K, SADAMU N AND YOSHIHIRO U. (1971). Automated Feulgen's reaction in autoscreening. *J. Jpn. Soc. Clin. Cytol.*, **10**, 148–154.

MOUTAIN CF. (1986). A new international staging system for lung cancer. *Chest*, **4**, 225s–233s.

OPFERMANN M, BRUGAL G AND VASSILAKOS P. (1987). Cytometry of breast carcinoma: Significance of ploidy balance and proliferation index. *Cytometry*, **8**, 217–224.

PENCE JC, KERNS BM, DODGE RK AND IGLEHART JF. (1993). Prognostic significance of the proliferation index in surgically resected non-small cell lung cancer. *Arch. Surg.*, **128**, 1382–1390.

SALVATI F, TEODORI L, GAGLIARDI L, SIGNORA M, AQUILINI M AND STORNIELLO G. (1988). DNA flow cytometric studies of 666 human lung tumours analyzed before treatment. *Chest*, **96**, 1092–1098.

SIMONY J, PUJOL JL, RADAL M, URSULE E, MICHEL FB AND PUJOL H. (1990). *In situ* evaluation of growth fraction determined by monoclonal antibody Ki-67 and ploidy in surgically resected non-small cell lung cancers. *Cancer Res.*, **50**, 4382–4387.

SMITH L, BARLOGIE B AND ALEXANIAN R. (1986). Biclonal and hypodiploid multiple myeloma. *Am. J. Med.*, **80**, 841–843.

SOBIN LH, HERMANEK P AND HUNTER RVP. (1987). *TNM Classification of Malignant Tumours*. 4th ed. UICC: Geneva.

THEUNISSEN PHMH, LEERS MPG AND BOLLEN ECM. (1992). Proliferating cell nuclear antigen (PCNA) expression in formalin-fixed tissue of non-small cell lung carcinoma. *Histopathology*, **20**, 251–255.

TISI GM, FRIEDMAN PJ, PETERS RM, PEARSON G, CARR D, LEE RE AND SELAWRY O. (1982). American Thoracic Society: clinical staging of primary lung cancer. *Am. Rev. Respir. Dis.*, **125**, 659–664.

TUBIANA M AND COURDI A. (1989). Cell proliferation kinetics in human solid tumors: relation to probability of metastatic dissemination and long-term survival. *Radiother. Oncol.*, **15**, 1–18.

TUNGEKAR MF, GATTER KC, DUNNILL MS AND MASON DY. (1991). Ki-67 immunostaining and survival in operable lung cancer. *Histopathology*, **19**, 545–550.

VOLM, M, DRINGS P, MARRTERN J, SONKA J, VOGT-MOYKOPF I AND WAYSS K. (1985). Prognostic significance of DNA patterns and resistance predictive tests in non-small cell lung carcinoma. *Cancer*, **56**, 1396–1403.

WORLD HEALTH ORGANIZATION. (1982). The World Health Organization histological typing of the lung tumors. 2nd ed. *Am. J. Clin. Pathol.*, **77**, 123–136.

ZIMMERMANN PV, HAWSON GAT, BINT MH AND PARSONS PG. (1987). Ploidy as a prognostic determinant in surgically treated lung cancer. *Lancet*, **2**, 530–533.